# Role of Lymphocytic Choriomeningitis Virus (LCMV) in Understanding Viral Immunology: Past, Present and Future

**DOI:** 10.3390/v4112650

**Published:** 2012-10-29

**Authors:** Xin Zhou, Srividya Ramachandran, Margaret Mann, Daniel L. Popkin

**Affiliations:** 1 Department of Dermatology, Case Western Reserve University, 10900 Euclid Avenue, Cleveland, OH 44106, USA; Email: xin.zhou@case.edu (X.Z.); sxr476@case.edu (S.R.); margaret.mann@gmail.com (M.M.); 2 Department of Dermatology, Pathology, Microbiology & Molecular Biology, Case Western Reserve University, 10900 Euclid Avenue, Cleveland, OH 44106, USA

**Keywords:** lymphocytic choriomeningitis virus, immunology, viral immunology, immune memory, persistent infection

## Abstract

Lymphocytic choriomeningitis virus (LCMV) is a common infection of rodents first identified over eighty years ago in St. Louis, MO, U.S.A. It is best known for its application in immunological studies. The history of LCMV closely correlates with the development of modern immunology. With the use of LCMV as a model pathogen several key concepts have emerged: Major Histocompatibility Complex (MHC) restriction, T cell memory, persistent infections, T cell exhaustion and the key role of immune pathology in disease. Given the phenomenal infrastructure within this field (e.g., defined immunodominant and subdominant epitopes to all T cell receptor specificities as well as the cognate tetramers for enumeration *in vivo*) the study of LCMV remains an active and productive platform for biological research across the globe to this day. Here we present a historical primer that highlights several breakthroughs since the discovery of LCMV. Next, we highlight current research in the field and conclude with our predictions for future directions in the remarkable field of LCMV research.

## 1. Introduction

**Figure 1:**
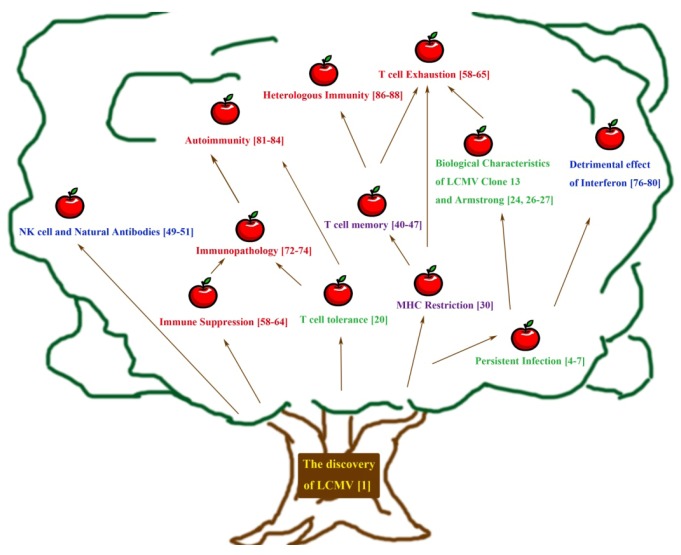
Tree of knowledge as disseminated by the lymphocytic choriomeningitis virus (LCMV) model system. This tree depicts a partial history of significant research milestones with the LCMV model system. The conceptual and/or experimental relationship between different discoveries is represented by uni-directional arrows. Green font is used to represent the first part of this review, concepts in persistent viral infection. Purple is used to highlight the second section, T cell immunity. Blue is used to represent the third section, innate immunity. Lastly, red represents the fourth and final section of immune dysfunction. Of note, certain scientific ideas (e.g., “T cell exhaustion”) are conceptually and experimentally derived from multiple lines of research and thus relevant to multiple sections of this review and our understanding of viral immunology. However, for simplicity they are represented by a single color.

Both innate and adaptive immune systems have evolved to protect the host from pathogens. By studying pathogens and how they interact with the host, many fundamental concepts of the immune system have been elucidated. In this review we will discuss how studies with lymphocytic choriomeningitis virus (LCMV), a natural pathogen of mice, contributed toward our understanding of immunology. Key concepts such as persistent viral infection, MHC restriction, and T cell exhaustion were discovered from the study of LCMV. This review will cover the origin of LCMV and highlight significant milestones along its history up to present day with a focus on fundamental discoveries pertaining to immunology and viral pathogenesis (summarized in Figure 1, a graphical outline of this review). Then, we will speculate on the future of LCMV research given its rich history towards understanding viral immunology. 

## 2. Early Approaches towards Understanding Persistent Infection and Its Immune Response

Lymphocytic Choriomeningitis Virus (LCMV) was isolated serendipitously by Charles Armstrong when he studied epidemic encephalitis in St. Louis in 1933 [1,2]. While trying to determine the virus responsible for this epidemic, Armstrong isolated LCMV, which he named secondary to host lymphocytosis and inflammation in the meninges lining the brain (including the choroid plexus lining the intracerebral ventricles) and spinal cord. Interestingly, LCMV was uncovered as it is a common pathogen in the house mouse and thus was present in the field. Ultimately, the infectious etiology of the St. Louis encephalitis epidemic was the flavivirus St. Louis encephalitis virus [3]. Although Armstrong was credited with the discovery of LCMV, other investigators also studied this virus early in its modern history. Traub and Rivers [4,5,6,7] published studies with LCMV in mouse and human in 1936 making observations towards both the virus and the immune response. 

One such topic was the observation of persistent *versus* acute viral infections. Previously, it was thought that a virally infected host either succumbed to the infection or cleared the infection within a short period of time. In contrast, Traub in 1936 found that mice infected with LCMV in utero or shortly after birth neither die nor eliminate the virus [5]. Burnet and Fenner proposed that these newborn mice were persistently infected with LCMV because they were “immunologically tolerant”. By virtue of being born from an infected environment (*i.e.*, the mother) [8], the newborn’s immune system was “tolerant” towards the virus and regarded it as “self” as opposed to a “non-self” pathogen. Supporting this idea, antibodies to LCMV virus were not detected in these persistently infected mice [9]. 

However, the beginning of many striking turns in the history of LCMV occurred shortly thereafter. With the advent of more sensitive radio immune techniques, Oldstone and Dixon were able to detect low levels of antibody in complex with LCMV in the kidney [10,11]. Therefore, an immune response was present. The absence of circulating antibodies was due to its accumulation as immune complexes in the glomeruli [10,12]. These findings were challenged when it was appreciated that an immune response as measured by antibody production was dependent on both the strain of mouse and virus [13,14,15,16,17,18,19].

In particular, this strain dependence was first uncovered when Volkert *et al.* observed a different result from Oldstone & Dixon in 1975. This group could not detect any antibodies in the peripheral blood nor the glomeruli in AKR mice infected by LCMV-Traub, using either ordinary serological methods or immunofluorescence techniques [20]. Oldstone *et al.* then directly addressed this possibility of strain dependence by infecting several different lines of mice: SWR/J, CBA/H WEHI, BALB/H WEHI as well as their hybrid offspring. They found that the SWR host had the strongest antibody response and BALB had the weakest antibody response. During this process, they found that C1q bound complexes, which were correlated with the production of antibodies, was controlled by the H-2 (or histocompatibility) complex, another example of the intimate connection between viral pathogenesis and immunology during the study of LCMV [21,22]. Furthermore, in 1991 this same group confirmed that virus strain also plays a role by demonstrating that the antibody response of SWR/J mice infected with LCMV Armstrong is 5–10 times higher than when these same mice were infected with LCMV Traub [23], illustrating the importance of viral variation. 

The genetic variation in viral isolates can also account for variations in viral persistence. Although well appreciated now for several viruses this was demonstrated using LCMV infection as an experimental model in the early days of experimental virology. Hotchin *et al.* found the passage of virus can affect persistence of LCMV infection [14] which was related to the immune response. After 10 passages, virus titers from the liver were ~20 times higher than titers from brain. Also, newborn mice infected with viral isolates passed from liver were more likely to die than the strains passed from brain. In 1984, Ahmed *et al.* studied the functional consequence of LCMV isolates by observing the virological outcome after infecting mice with virus isolated from different organs (e.g., brain and spleen). These results showed that LCMV which originates from brain continues to have a central nervous system (CNS) tropism, results in a brisk and fulminant cytotoxic T lymphocyte (CTL) response and is subsequently cleared rapidly by the host. In striking contrast, viruses isolated from spleen are tropic for visceral organs, potentiate less vigorous CTL responses and ultimately can exist in recipient mice indefinitely. One of the best characterized examples of this is the variant known as “Clone 13”. LCMV Clone 13 was a variant of the parent Armstrong virus which was isolated from the spleen of carrier mice which sustained a persistent LCMV infection since birth. Clone 13 was one of many splenic clones which could suppress LCMV-specific CTL responses [24]. Infection with Clone 13 significantly alters the tissue distribution of virus as well as the immunodominance hierarchy of LCMV-specific CD8 T cells compared to Armstrong [25]. Clone 13 was chosen for further characterization as its profound biological phenotype of persistence (in contrast to the neurotropic and acutely cleared LCMV Armstrong) occurred, despite it having the least amount of genetic variation among many clones isolated with similar biological activity.

With the development of modern sequencing techniques, investigators sequenced and uncovered that the Clone 13 and Armstrong virus genomes differed by only 5 out of 10,600 nucleotides [26]. Subsequently, Matloubian *et al.* demonstrated that only two amino acid differences between Armstrong and Clone 13 were critical—a K→Q substitution at position 1079 and F→L substitution at position 260. These two differences in the viral “polymerase” and “glycoprotein” genes, respectively, accounted for the biological phenotype of acute *versus* persistent infection. It also correlated with several other biomarkers such as the virus yield per macrophage and number of macrophage being infected [27]. This concept that single amino acid changes in a pathogen can dramatically alter the *in vivo* persistence of a virus was first clarified using LCMV. This principle has been extended by studies in other related fields such as HIV [23], hepatitis E virus [28] and potentially autoimmunity [29].

## 3. Adaptive Immunity: Understanding the T Cell Mediated Immune Response Using LCMV

In this section, we review T cell mediated immune responses in 3 parts: T cell recognition of its target via MHC restriction, leading to perforin mediated killing of this target and finally durable memory as a result of this process.

**Figure 2:**
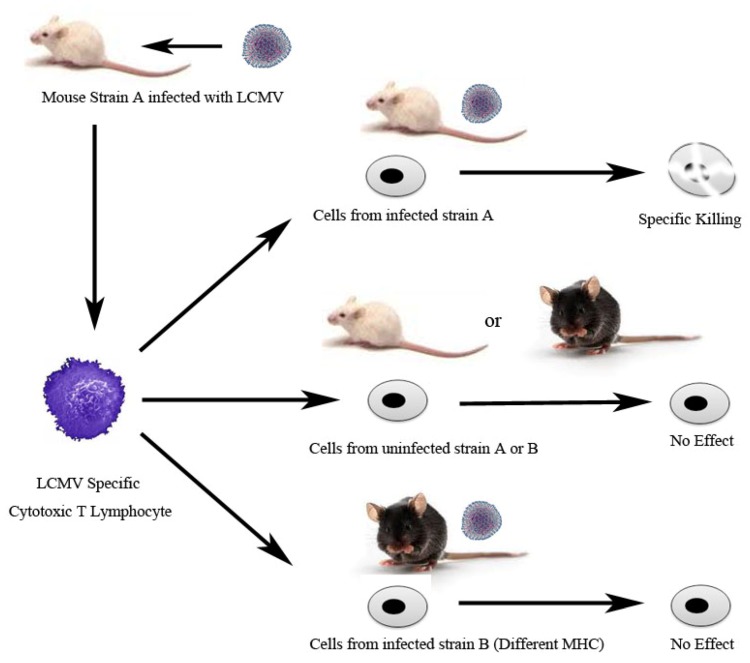
The Discovery of Major Histocompatibility Complex (MHC) restriction. These experiments were completed and published in 1974. Rolf Zinkernagel and Peter Doherty harvested splenocytes containing viral specific cytotoxic T lymphocytes (CTL) from infected mouse strain A. These CTL were then mixed *in vitro* with either C3H mouse fibroblasts or macrophages derived from different strains, infected with LCMV or uninfected, and labeled with Chromium-51 (Cr-51). CTL killing of target cells was assessed by release of Cr-51. These experiments demonstrated that LCMV specific CTL are “MHC restricted” and thus only lyse cells carrying the same MHC as the strain generated from those CTL [30].

One of the fundamental tenets of the adaptive immune response, MHC restriction, came about from the Nobel Prize winning studies performed by Zinkernagel and Doherty with LCMV. Prior to this, the MHC locus was studied exclusively in transplantation and tumor rejection. Zinkernagel and Doherty did several experiments to address how the MHC genetic locus contributed to the CTL response against LCMV infected cells [30,31,32]. These studies demonstrated the now appreciated as more conventional and ubiquitous role for cytotoxic T lymphocytes to kill their MHC restricted targets. Specifically, this work demonstrated that the cellular immune system simultaneously recognizes both the foreign peptide as well as the major histocompatibility antigens. This made clear the role of the MHC in the normal immune response. Their key experiment was as follows: LCMV specific CTL from infected strain A were mixed with cells from 2 different mouse strains, A and B. They observed that CTL killed only cellular targets from the LCMV infected strain A. The CTL did not lyse uninfected cells from either strain A or B nor infected cells from strain B (Figure 2). They concluded that CTL can only recognize foreign antigen in the context of the originating MHC to trigger lysis of the MHC matched target. Subsequently, Zinkernagel and Doherty went on to demonstrate that this restriction of CTL was dependent on the specific MHC environment in the thymus from which they originated [33]. 

These studies were confirmed and extended by other groups. That is, it was subsequently observed that this CTL response was critical to clear an LCMV infection [34] and that consistent with this idea, the transfer of cloned CTL was sufficient to rapidly clear an otherwise persistent LCMV infection [35]. 

These experiments demonstrated a critical missing link in the field of immunology—how a T cell is able to recognize its target under conventional physiologic conditions. This has vast implications throughout immunology and medicine including infectious diseases, tumor immunotherapy and autoimmunity. In order to understand how infections are cleared and augment this process, eliminate tumors with T cells or to understand allergies and autoimmunity, the fundamental rules governing T cell recognition of its target provides a necessary foundation for our understanding and thus approach to this day. Specifically, since these landmark findings and to date, investigators have observed that the human leukocyte antigen (HLA) locus in human (MHC in mice) is the most highly associated genetic locus with human disease. Current genome wide association studies (GWAS) reinforce this biological fact that time and time again, the HLA locus is the most critical determinant towards susceptibility *versus* resistance to autoimmune or infectious diseases. This is largely attributed to the critical function of the HLA to educate and direct T cell activity—notably uncovered by infecting mice with LCMV and determining that the T cell response was restricted by the MHC.

In addition to the discovery of MHC restriction, the study of LCMV also contributed to the understanding of T cell mediated cell lysis, in particular perforin based cytotoxicity [36]. Kagi *et al.* generated perforin deficient mice and found that T cell mediated cytotoxicity to LCMV-WE infected cells were largely absent in these mice [37]. Additionally, these mice were not able to eliminate MHC class I positive MC57G fibrosarcoma tumor cells, indicating that functional CTLs and Natural Killer (NK) cells were not present in these perforin deficient mice.

A key feature of the adaptive immune response is memory. Studies with LCMV played a key role in understanding this process. T cells “remember” their cognate antigen after initial antigen encounter. After a period of complete absence of antigen, subsequent antigen encounters are noted by a more rapid and potent response. Although now widely accepted, formal proof of this concept was acquired in part by extensive studies in the LCMV model system. LCMV was and continues to be an ideal model to study immunological memory for three primary reasons: (1) LCMV Armstrong is a natural acute infection in mice, *i.e.*, the virus is completely eliminated by its host in a very short period of time after infection, ~1 week. (2) Critical reagents such as T-cell receptor (TCR) transgenic mice for both CD4 and CD8 T cells are readily available. Additionally the cognate MHC tetramers to detect these T cells via conventional flow cytometry were obtained early on for the LCMV model system. These reagents allow the investigator to easily enumerate endogenous viral antigen specific T cell responses [38]. (3) The T cell response to LCMV is very robust. As this response is relatively large and focused, it allows the investigator to more easily assess T cell responses accurately. Findings gained from experimental LCMV research have been validated in a pioneering study that followed yellow fever and small pox-specific T cell responses in humans [39]. These important features have allowed several labs to make significant contributions towards our understanding of T cell memory. A thorough discussion of these findings is beyond the scope of this review. Therefore, we will focus on specific key contributions to CD8 T cell memory. Maintenance of memory in the absence of antigen is a critical feature of memory [40,41]. In the study by Lau *et al*., the investigators adoptively transferred LCMV immune CD8 T cells into uninfected recipient mice and then performed a second transfer 18 months after the original transfer. They then used many sensitive immunological, viral and molecular assays to show that LCMV was absent from the purified CD8 T cells. At this point, the investigators quantitated durable antigen specific memory responses over time and showed that this memory (after 26 months, greater than the average mouse lifespan) was still protective against a virulent LCMV challenge. 

**Figure 3:**
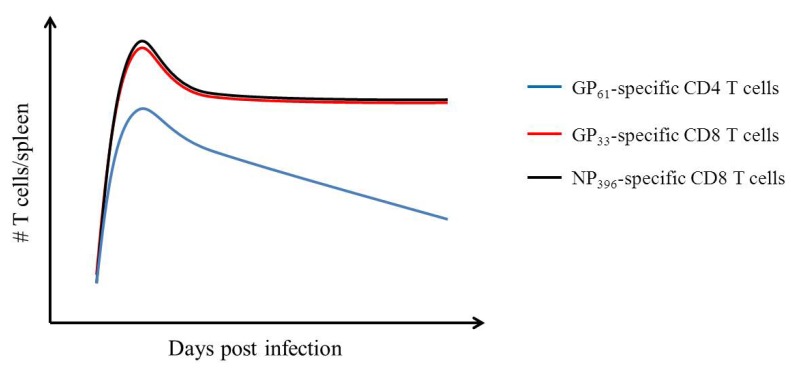
Schematic of epitope-specific CD4 and CD8 T cell response from acute activation to memory. Data adapted from Homann *et al.* 2001 [44]. Splenocytes were collected on the day post infection indicated. MHC tetramer staining was performed to enumerate T cells specific to the LCMV GP61 epitope- (CD4 T cell specific; blue line) as well as the LCMV CD8 T cell specific epitopes, GP33 (red line) and NP396 (black line).

Another property of memory is the expansion and maintenance of a memory pool from a smaller naïve precursor population. Characterization of this kinetic expansion of naive CD8 T cells precursors was performed using LCMV Armstrong. Here investigators tracked LCMV antigen specific T cells using tetramerized MHC molecules labeled with a fluorophore. They concluded that there is 1 precursor specific for the LCMV immunodominant H-2Db-restricted GP33-41 epitope for every 2 × 10^5^ CD8 T cell in the naïve animal [42]. Those precursors can divide ~14 times during the first week of infection, resulting in a >16,000 fold expansion. The effector T cell populations, after resolution of infection, underwent 2 different outcomes. Most of the effector T cells were eliminated via apoptosis; the rest of them become memory T cells. The apoptosis of effector T cells is termed the “contraction phase”. The contraction of effector T cells may be via co-regulation of the IL-2 family, TNF-family members, perforin and IFN-γ [43]. The total number of antigen specific T cells contracted ~10 fold after the peak of expansion [43]. Subsequent to T cell contraction, CD8 memory T cells form a stable pool, ~10^6^ per spleen, whereas CD4 T cell numbers slowly erode (Figure 3). This was determined by following viral antigen-specific T cells over ~3 years using tetramers to the CD8 T cell epitopes (GP33, GP276, GP118, GP92, NP396, NP205) and CD4 T cell epitopes (GP61, NP309). It was observed that the CD8:CD4 ratio increases from 10:1 twenty dpi to 100:1 at 900 dpi [44].

Memory T cells can be divided into two subsets, central memory and effector memory [45]. In 2003 the lineage relationship of those two subsets was described by Ahmed’s group using LCMV and *Listeria monocytogenes*. They observed a conversion from T cell effector memory to the central memory population [46]. This remains a controversial field. One example is that the repertoires of circulating human CD8 central and effector memory T cell subsets appear to be distinct in contrast to the mouse system [47].

## 4. Understanding Innate Immunity: Contributions from the LCMV System

LCMV also contributed to the understanding of the innate immune system, the first barrier of the body to pathogens. Natural Killer cells, or NK cells, were introduced in 1972 by Evans for its selective tumor killing characteristics [48]. Welsh *et al.* in 1977 [49], found that after LCMV infection, early cytotoxic cells (ECC) were generated one day post infection in spleen and peritoneum. These ECC emerge and kill both infected and uninfected cell lines and their peak activity appears at three day post infection [50]. On the other hand, the adaptive immune response began around five days post infection. Ray Welsh’s lab continues to follow the role of NK cells during LCMV infection. Their most recent studies have implicated NK cells as master regulators of CD4 T cells which subsequently control CD8 T cells during viral infections [51]. These findings provide further evidence for the importance of NK cells as innate effectors and an essential bridge in directing an appropriate adaptive immune response.

The study of LCMV also contributed to our knowledge of natural antibodies. Natural antibodies were considered “background antibody” since they constitute only ~2%–10% of the total serum in naive mice and had no clear biological role. The functional role of natural antibody was and still is poorly understood. However, one potential role of natural antibodies demonstrated by the Zinkernagel lab was in the LCMV antiviral response [52]. This study showed that natural antibody acts to “redistribute” viral antigen. Specifically, virus in the circulation is bound to natural antibody which enhances virus uptake by the secondary lymphoid organs. Since secondary lymphoid organs are widely accepted as the crucible to initiate cellular immunity [53], this is likely to be advantageous to protect the host from pathogen challenge. In contrast, in the absence of natural antibody, virus was found in end organs such as liver and brain which may then suffer directly from viral infection or their sequelae. 

## 5. Immune Dysfunction as Observed Using the LCMV Model — Immunosuppression, Immune Exhaustion, Immunopathology, Autoimmunity and Heterologous Immunity

Like other complex systems, the immune system is susceptible to abnormal or impaired functioning. The immune system has almost certainly evolved to protect us from morbidity and mortality dealt by infectious microorganisms. However, it is now well appreciated that this system can be incorrectly silenced, falsely activated and/or wrongly directed. The concept and experimental evidence for immune dysfunction originated in part from studies with LCMV. Conceptually, these findings were key to better understanding the range of disease which may emanate from a system which evolved to protect the host as well as better understanding methods to modulate an immune response therapeutically. 

Immunosuppression is one example of immune dysfunction. It was appreciated early on that measles can cause immunosuppression [54] which is likely due to virus modulation of dendritic cell function and development [54]. Similarly, it was later shown that LCMV is capable of initiating immunosuppression [55]. The LCMV system provided several unique features beyond what was observed prior. In contrast to lytic viruses which directly infect lymphocytes to induce immunosuppression, LCMV is a non-lytic virus and primarily infects non-lymphocytic cells. Despite this, LCMV infected animals were unable to generate a robust cytotoxic T lymphocyte (CTL) response [56]. Concomitant with these studies was the finding that LCMV clones which persistently infected animals resulted in immunosuppression (including decreased CTL activity and thymocyte number whereas those viral clones which were acutely cleared did not [24,57]). These studies serve as a basis for understanding how individuals become immunosuppressed during viral infection. Moreover, one can harness these mechanisms to therapeutically suppress the immune response. 

A second aspect of immune dysfunction is the phenomenon of “immune exhaustion” which may also be considered a subtype of virus induced immune suppression. Capitalizing on isolates of persistent (*versus* acute) LCMV which had been previously defined, the mechanism of persistent infection has been studied extensively using Clone 13 and Docile LCMV variants. In 1993, Moskophidis *et al.* introduced the concept of “exhaustion” of effector T cells during a persistent LCMV infection, in which they coined the term. They found a defect in both antigen specific T cell function and number which correlated with a viremia of 10^4^ PFU/mL or higher [58]. Exhausted virus‑specific CD8 T cells, despite being highly activated, also demonstrate upregulated inhibitory signals which diminish their effector functions [59,60]. This exhaustion or loss of T cell function is thought to be caused by continuous high level stimulation of T cells. Consistent with this idea, Mueller and Ahmed observed that decreasing MHC Class I stimulation of CD8 T cells (with an MHC I −/− environment using bone marrow chimeric mice) resulted in increased numbers of polyfunctional T cells during a persistent LCMV Clone 13 infection [61] (polyfunctionality referring to the extensive production of multiple cytokines by an individual T Cell, a paradigm established in part by LCMV). This phenomenon of immune exhaustion is generalizable and observed with other infections such as HIV, Hepatitis B and Hepatitis C [62,63,64]. Interestingly, with further investigations into the mechanism of T cell exhaustion by multiple inhibitory pathways, we now appreciate a more complex set of cues to resurrect T cell function (e.g., by blocking the PD-1, IL-10, TGF-beta and LAG-3 pathways in addition to augmenting IL-7 signaling [65,66,67,68,69,70,71]).

A third form of immune dysfunction is immunopathology in which disease is associated with or directly caused by the immune system and not as a direct result of the pathogen burden or toxicity from the infected cell. Immunopathology is now appreciated to happen fairly often and largely in the context of infection. Therefore by suppressing the immune response, one can then eliminate the clinical disease, even when the viral infection is not treated. An early example in the LCMV literature was the development of LCMV meningitis which was not due to direct viral infection of the central nervous system (CNS) but collateral CTL dependent damage [72]. Consistent with this idea, observations from Nathanson’s group reported during the same year, found that in the immunosuppressed mouse no CNS disease was observed [55,56,73,74]. These results provided a dramatic example of how the immune response and not direct infection of virus was responsible for clinical disease. Recently, this CTL-dependent immunopathology has been demonstrated to be secondary to a myelomonocytic cell recruitment. Specifically, investigators used multiphoton microscopy and antibody depletion to show that macrophages and neutrophils are the key effector cells, (directed by CTL) causing fatal CNS vascular injury during acute LCMV meningitis [75]

LCMV also contributed to the understanding of interferon in immunopathology. Interferon is a protein well known to virologists as the premier anti-viral cytokine. Much of this work is secondary to the development of a neutralizing anti-sera produced in sheep. Using this reagent, investigators demonstrated the essential anti-viral role of interferon *in vivo* [76,77]. However, as earlier as 1975 these investigators also demonstrated that interferon may have a detrimental effect. Mice injected with interferon daily after birth underwent liver degeneration, glomerulonephritis and death [78,79]. Using this sheep anti-sera to deplete interferon, the deleterious effects of LCMV in the congenitally infected mouse model were also shown to be interferon dependent [80]. Consistent with the central idea of immunopathology, Riviere *et al.* found that depletion of interferon in LCMV infected mice inhibited weight loss, liver cell necrosis and death, despite an ~100 fold increase in viremia [80].

Another form of immune dysfunction is autoimmunity. In an early example of immune reactivity to self, Pfizenmaier *et al.* found that LCMV stimulated lymphocytes attacked both infected target cells and noninfected syngeneic target cells between 4 and 6 days post infection. As the infection progressed, the immune response was recognized to be more specific to LCMV infected cells [81,82]. This early self-reactive immune response was considered autoimmunity, *i.e.*, a dysfunction in self *versus* non-self discrimination. In a focused effort to establish models of virus induced autoimmunity, two groups independently setup models of LCMV induced diabetes by expressing the LCMV glycoprotein (GP) under the control of the rat insulin promoter. This directed expression of LCMV GP as a “self” antigen in the beta islet cells of the pancreas [83,84]. In this way, these groups established small animal models in which autoimmunity could be triggered by viral infection. Upon infection, “self-reactivity” would occur and testable hypotheses regarding the mechanism of viral induced autoimmunity could be more easily addressed. A similar model in the CNS was later established whereby viral antigen was transgenically expressed in oligodendrocytes resulting in CNS disease only after viral infection [85]. These models have allowed investigators to assess the concept of “molecular mimicry” as a potential source of autoimmunity triggered by infection. Given the potential amino acid sequence similarities between host and pathogen, molecular mimicry may be the basis for T or B cell cross-reactivity between host and pathogen. 

**Figure 4:**
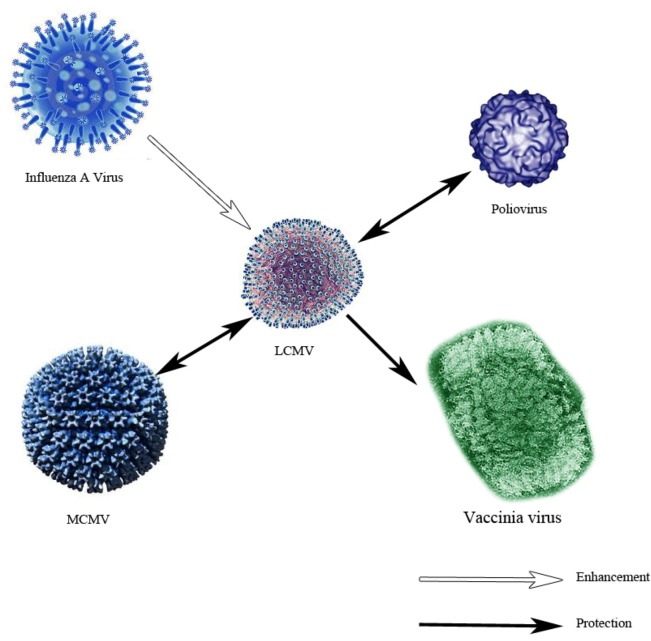
Heterologous immunity. This figure shows the relationship of heterologous immunity between LCMV and other viruses. These experiments were performed using C57BL/6 mice. The black filled arrows indicate a protective effect between the first infection (base of arrow) and the challenge infection (head of arrow). In some cases this protection from heterologous immunity is bi-directional as shown. On the contrary, the unfilled arrow depicts an enhancement of disease when infection of influenza A precedes LCMV infection.

One final potential source of immune dysfunction is from heterologous immunity. At its most basic, heterologous immunity stems from the observation that infection by one pathogen may protect an individual from a phylogenetically distinct invader. In some ways similar to “molecular mimicry” the mechanism of heterologous immunity is thought to be from a cross-reactive lymphocytic response. LCMV infection affords subsequent protection towards vaccinia virus [86] by increased CTL responses toward vaccinia virus infection quantitated by IFNγ production [87]. Another variation of this is observed when infection with either the arenavirus Pichinde virus or vaccinia virus activated the CTL response specific to LCMV (Figure 4) [88]. These findings are reviewed in greater depth elsewhere [89]. Although recognized as a potential for increased immune protection, heterologous immune responses can also be devastating for the host. A dramatic and clinically important example is the potentially lethal outcome when individuals are sequentially infected with different serotypes of dengue virus. The pathogenesis of dengue hemorrhagic fever may involve the amplification of inferior but cross-reactive T cell clones. In the complex real-world situation of multiple pathogen encounters, altering the hierarchy of epitope-specific T cell clones results in largely unpredictable outcomes. The use of model systems with well characterized viruses such as LCMV is key for determining incremental understanding to piece together this complex puzzle. 

## 6. From the Present and Onwards

As an established model system, LCMV will continue to contribute to the field of viral immunology. We envision these contributions to fall in two major categories—the study of persistent viral infections and vaccine development.

Regarding the study of persistent viral infection, LCMV is noteworthy as the best characterized platform to study acute *versus* persistent viral infection side by side in its natural host Mus musculus which is also the premier genetic system for mammalian genetics. This is largely because of the profound phenotypic difference between LCMV Armstrong and its variant Clone 13 which results from only a two nucleotide difference. This genetic conservation allows the investigator to have great focus in being able to assign function without being lost in the complexity of most viral genotype to phenotype searches. Having said that, it is still unclear exactly how either of these mutations confer persistence. We know that both mutations are needed. The glycoprotein mutation affects tropism and the polymerase affects replicative capacity [90,91]. How these mutations and their altered function mechanistically confer their observed activity is unknown. Moreover how this activity translates into such a profound difference physiologically (acute *versus* persistent infection) is actively being studied but not completely understood. We suspect that these advances will continue to be useful in understanding some of the shared patterns between all persistent viral infections. Towards this point, the genetic similarities of these two viral variants has left the immunodominant epitopes untouched and resulted in numerous studies characterizing acute *versus* persist viral infections side by side and with a minimum of confounding factors. 

We foresee these studies providing the field with great insight towards the ever increasing burden of persistent viral infection in our modern society. Importantly, cancer in many ways mimics persistent viral infection as a parasitic entity which evades and progresses in spite of the body’s attempts to eliminate it. Thus studies with LCMV will likely help us better understand cancer as well. Consonant with this is one of the great modern successes in LCMV studies—that interrupting the PD-1 pathway can resurrect an otherwise exhausted immune response. This concept of uncoupling the immune response from negative signaling pathways has been applied successfully to the treatment of numerous tumors using monoclonal antibodies to both PD-1 and PD-L1 in humans (published during the writing of this review [92,93]. PD-1 antibody treatment has also been used to enhance SIV-specific immunity *in vivo*, in macaques and to reverse immune dysfunction of HIV-specific T cells *in vitro* from infected donor samples [94,95]. Because of the large and focused immune response to LCMV, it has been a strong model system for T cell memory and the related field of vaccinology. Indeed, many of the fundamental rules of memory were originally investigated with the CD8 T cell response towards LCMV. This work is now being applied to a refined mechanistic understanding of CD4 T cell memory [96]. Because of its intense use as a platform for CD8 T cell memory, LCMV was recently used to quantitate the efficacy of a novel hydrogen peroxide-based vaccine platform [97]. Interestingly, LCMV is itself being used as a vector platform for vaccination [98,99]. Therefore, we anticipate that from many different approaches, the LCMV system will be a key player in our future refinement of vaccinology. This is critical as vaccines are widely considered to be the most efficient use of health care funds to alleviate morbidity and mortality worldwide.

In addition to a primary focus on the above (persistent viral infections and vaccines), we expect that some energy will be focused on the biology of this arenavirus itself. This is largely because of its recognition as a cause of meningitis in the growing immunosuppressed population [100,101,102,103] and as the prototype of a family of more lethal human hemorrhagic fever viruses. The most discussed of these is Lassa fever (endemic in sub-Saharan Africa). Other strains are endemic in South America (e.g., Junin and Machupo viruses). And new strains causing significant morbidity and mortality continue to be recognized (e.g., Lujo in southern Africa). Importantly, as our world continues to become more interconnected, these lethal viral infections will likely enter further regions of the globe. For these reasons, the United States National Institute for Allergy and Infectious Diseases (NIAID) has deemed much of the arenaviridae to be “Category A Priority Pathogens”. This is the highest level of importance for the high potential for morbidity and mortality from an infectious agent which is relatively easy to produce and transmit. Indeed, with the advent of reverse genetic techniques for arenaviruses pioneered by the de la Torre group [104,105,106,107,108,109,110] the production of designer arenaviruses is now almost routine.

Although we are familiar with the disclaimer “past performance is not a guarantee of future results”, we suspect that future studies with LCMV will be even more productive than its illustrious past. As biomedical research continues to become more complex, the rich infrastructure of the LCMV model system will be key. It provides not only reductionist approaches but also enough plasticity to clarify a wide variety of questions in viral immunology and beyond. We believe the multitude of reagents and large body of experimental findings will provide experimental anchors and handles to guide future investigators along the ever increasingly complex road of biomedical research.

## References

[1] Muckenfuss R.S., Armstrong C., Webster L. (1934). Etiology of the 1933 epidemic of encephalitis. J. Am. Med. Assoc..

[2] Beeman E.A. (2007). Charles Armstrong MD: A Biography.

[3] Luby J.P.  (1979). St. Louis encephalitis. Epidemiol. Rev..

[4] Traub E. (1936). An epidemic in a mouse colony due to the virus of acute lymphocytic choriomeningitis. J. Exp. Med..

[5] Traub E. (1936). Persistence of lymphocytic choriomeningitis virus in immune animals and its relation to immunity. J. Exp. Med..

[6] Rivers T.M., Scott T.F. (1936). Meningitis in man caused by a filterable virus: II. Identification of the etiological agent. J. Exp. Med..

[7] Traub E. (1936). The epidemiology of lymphocytic choriomeningitis in white mice. J. Exp. Med..

[8] Burnet F.M., Fenner F. (1949). The Production of Antibodies.

[9] Weigand H., Hotchin J. (1961). Studies of lymphocytic choriomeningitis in mice. J. Immunol..

[10] Oldstone M.B., Dixon F.J. (1967). Lymphocytic choriomeningitis: Production of antibody by "tolerant" infected mice. Science.

[11] Hotchin J. (1971). The contamination of laboratory animals with lymphocytic choriomeningitis virus. Am. J. Pathol..

[12] Benson L., Hotchin J. (1969). Antibody formation in persistent tolerant infection with lymphocytic choriomeningitis virus. Nature.

[13] Rowe W. (1954). Studies on pathogenesis and immunity in lymphocytic choriomeningitis infection of the mouse. Rev. Rep. Naval Med. Res. Inst..

[14] Hotchin J., Benson L.M., Seamer J. (1962). Factors affecting the induction of persistent tolerant infection of newborn mice with lymphocytic choriomeningitis. Virology.

[15] Hotchin J. (1962). The biology of lymphocytic choriomeningitis infection: Virus-induced immune disease. Cold Spring Harb. Symp. Quant. Biol..

[16] Hotchin J., Benson L. (1963). The pathogenesis of lymphocytic choriomeningitis in mice: The effects of different inoculation routes and the footpad response. J. Immunol..

[17] Oldstone M.B., Dixon F.J. (1968). Susceptibility of different mouse strains to lymphocytic choriomeningitis virus. J. Immunol..

[18] Lehmann-Grube F., Slenczka W., Tees R. (1969). A persistent and inapparent infection of L cells with the virus of lymphocytic choriomeningitis. J. Gen. Virol..

[19] Lehmann-Grube F. (1964). Lymphocytic choriomeningitis in the mouse. I. Growth in the brain. Arch Gesamte Virusforsch.

[20] Volkert M., Bro-Jorgensen K., Marker O. (1975). Persistent LCM virus infection in the mouse. Immunity and tolerance. Bull. World Health Organ..

[21] Oldstone M.B., Buchmeier M.J., Doyle M.V., Tishon A. (1980). Virus-induced immune complex disease: Specific anti-viral antibody and C1q binding material in the circulation during persistent lymphocytic choriomeningitis virus infection. J. Immunol..

[22] Oldstone M.B., Tishon A., Buchmeier M.J. (1983). Virus-induced immune complex disease: Genetic control of C1q binding complexes in the circulation of mice persistently infected with lymphocytic choriomeningitis virus. J. Immunol..

[23] Tishon A., Salmi A., Ahmed R., Oldstone M.B. (1991). Role of viral strains and host genes in determining levels of immune complexes in a model system: Implications for HIV infection. AIDS Res. Hum. Retrovir..

[24] Ahmed R., Salmi A., Butler L.D., Chiller J.M., Oldstone M.B. (1984). Selection of genetic variants of lymphocytic choriomeningitis virus in spleens of persistently infected mice. Role in suppression of cytotoxic T lymphocyte response and viral persistence. J. Exp. Med..

[25] Wherry E.J., Blattman J.N., Murali-Krishna K., van der Most R., Ahmed R. (2003). Viral persistence alters CD8 T-cell immunodominance and tissue distribution and results in distinct stages of functional impairment. J. Virol..

[26] Salvato M., Borrow P., Shimomaye E., Oldstone M.B. (1991). Molecular basis of viral persistence: A single amino acid change in the glycoprotein of lymphocytic choriomeningitis virus is associated with suppression of the antiviral cytotoxic T-lymphocyte response and establishment of persistence. J. Virol..

[27] Matloubian M., Kolhekar S.R., Somasundaram T., Ahmed R. (1993). Molecular determinants of macrophage tropism and viral persistence: Importance of single amino acid changes in the polymerase and glycoprotein of lymphocytic choriomeningitis virus. J. Virol..

[28] Balint A., Farsang A., Zadori Z., Hornyak A., Dencso L., Almazan F., Enjuanes L., Belak S. (2012). Molecular characterization of feline infectious peritonitis virus strain DF-2 and studies on the role of ORF3abc in viral cell tropism. J. Virol..

[29] Tishon A., Oldstone M.B. (1987). Persistent virus infection associated with chemical manifestations of diabetes. II. Role of viral strain, environmental insult, and host genetics. Am. J. Pathol..

[30] Zinkernagel R.M., Doherty P.C. (1974). Restriction of *in vitro* T cell-mediated cytotoxicity in lymphocytic choriomeningitis within a syngeneic or semiallogeneic system. Nature.

[31] Zinkernagel R.M., Doherty P.C. (1974). Immunological surveillance against altered self components by sensitised T lymphocytes in lymphocytic choriomeningitis. Nature.

[32] Doherty P.C., Zinkernagel R.M. (1975). H-2 compatibility is required for T-cell-mediated lysis of target cells infected with lymphocytic choriomeningitis virus. J. Exp. Med..

[33] Zinkernagel R.M., Doherty P.C. (1975). H-2 compatability requirement for T-cell-mediated lysis of target cells infected with lymphocytic choriomeningitis virus. Different cytotoxic T-cell specificities are associated with structures coded for in H-2K or H-2D. J. Exp. Med..

[34] Buchmeier M.J., Welsh R.M., Dutko F.J., Oldstone M.B. (1980). The virology and immunobiology of lymphocytic choriomeningitis virus infection. Adv. Immunol..

[35] Byrne J.A., Ahmed R., Oldstone M.B. (1984). Biology of cloned cytotoxic T lymphocytes specific for lymphocytic choriomeningitis virus. I. Generation and recognition of virus strains and H-2b mutants. J. Immunol..

[36] Masson D., Tschopp J. (1985). Isolation of a lytic, pore-forming protein (perforin) from cytolytic T-lymphocytes. J. Biol. Chem..

[37] Kagi D., Ledermann B., Burki K., Seiler P., Odermatt B., Olsen K.J., Podack E.R., Zinkernagel R.M., Hengartner H. (1994). Cytotoxicity mediated by T cells and natural killer cells is greatly impaired in perforin-deficient mice. Nature.

[38] Murali-Krishna K., Altman J.D., Suresh M., Sourdive D.J.D., Zajac A.J., Miller J.D., Slansky J., Ahmed R. (1998). Counting antigen-specific CD8 T cells: A reevaluation of bystander activation during viral infection. Immunity.

[39] Miller J.D., van der Most R.G., Akondy R.S., Glidewell J.T., Albott S., Masopust D., Murali-Krishna K., Mahar P.L., Edupuganti S., Lalor S. (2008). Human effector and memory CD8+ T cell responses to smallpox and yellow fever vaccines. Immunity.

[40] Lau L.L., Jamieson B.D., Somasundaram T., Ahmed R. (1994). Cytotoxic T-cell memory without antigen. Nature.

[41] Murali-Krishna K., Lau L.L., Sambhara S., Lemonnier F., Altman J., Ahmed R. (1999). Persistence of memory CD8 T cells in MHC class I-deficient mice. Science.

[42] Blattman J.N., Antia R., Sourdive D.J., Wang X., Kaech S.M., Murali-Krishna K., Altman J.D., Ahmed R. (2002). Estimating the precursor frequency of naive antigen-specific CD8 T cells. J. Exp. Med..

[43] Kaech S.M., Wherry E.J., Ahmed R. (2002). Effector and memory T-cell differentiation: implications for vaccine development. Nat. Rev. Immunol..

[44] Homann D., Teyton L., Oldstone M.B. (2001). Differential regulation of antiviral T-cell immunity results in stable CD8+ but declining CD4+ T-cell memory. Nat. Med..

[45] Sallusto F., Lenig D., Forster R., Lipp M., Lanzavecchia A. (1999). Two subsets of memory T lymphocytes with distinct homing potentials and effector functions. Nature.

[46] Wherry E.J., Teichgraber V., Becker T.C., Masopust D., Kaech S.M., Antia R., von Andrian U.H., Ahmed R. (2003). Lineage relationship and protective immunity of memory CD8 T cell subsets. Nat. Immunol..

[47] Baron V., Bouneaud C., Cumano A., Lim A., Arstila T.P., Kourilsky P., Ferradini L., Pannetier C. (2003). The repertoires of circulating human CD8(+) central and effector memory T cell subsets are largely distinct. Immunity.

[48] Evans R., Alexander P. (1972). Mechanism of immunologically specific killing of tumour cells by macrophages. Nature.

[49] Welsh R.M., Zinkernagel R.M. (1977). Heterospecific cytotoxic cell activity induced during the first three days of acute lymphocytic choriomeningitis virus infection in mice. Nature.

[50] Welsh R.M. (1978). Cytotoxic cells induced during lymphocytic choriomeningitis virus infection of mice. I. Characterization of natural killer cell induction. J. Exp. Med..

[51] Waggoner S.N., Cornberg M., Selin L.K., Welsh R.M. (2012). Natural killer cells act as rheostats modulating antiviral T cells. Nature.

[52] Ochsenbein A.F., Fehr T., Lutz C., Suter M., Brombacher F., Hengartner H., Zinkernagel R.M. (1999). Control of early viral and bacterial distribution and disease by natural antibodies. Science.

[53] Karrer U., Althage A., Odermatt B., Roberts C.W., Korsmeyer S.J., Miyawaki S., Hengartner H., Zinkernagel R.M. (1997). On the key role of secondary lymphoid organs in antiviral immune responses studied in alymphoplastic (aly/aly) and spleenless (Hox11(-)/-) mutant mice. J. Exp. Med..

[54] McChesney M.B., Oldstone M.B. (1989). Virus-induced immunosuppression: Infections with measles virus and human immunodeficiency virus. Adv. Immunol..

[55] Mims C.A., Wainwright S. (1968). The immunodepressive action of lymphocytic choriomeningitis virus in mice. J. Immunol..

[56] McChesney M.B., Oldstone M.B. (1987). Viruses perturb lymphocyte functions: Selected principles characterizing virus-induced immunosuppression. Annu. Rev. Immunol..

[57] Thomsen A.R., Bro-Jorgensen K., Jensen B.L. (1982). Lymphocytic choriomeningitis virus-induced immunosuppression: Evidence for viral interference with T-cell maturation. Infect. Immun..

[58] Moskophidis D., Lechner F., Pircher H., Zinkernagel R.M. (1993). Virus persistence in acutely infected immunocompetent mice by exhaustion of antiviral cytotoxic effector T cells. Nature.

[59] Wherry E.J., Ha S.-J., Kaech S.M., Haining W.N., Sarkar S., Kalia V., Subramaniam S., Blattman J.N., Barber D.L., Ahmed R. (2007). Molecular signature of CD8+ T cell exhaustion during chronic viral infection. Immunity.

[60] Zajac A.J., Blattman J.N., Murali-Krishna K., Sourdive D.J.D., Suresh M., Altman J.D., Ahmed R. (1998). Viral immune evasion due to persistence of activated t cells without effector function. J. Exp. Med..

[61] Mueller S.N., Ahmed R. (2009). High antigen levels are the cause of T cell exhaustion during chronic viral infection. Proc. Natl. Acad. Sci. U. S. A..

[62] Khaitan A., Unutmaz D. (2011). Revisiting immune exhaustion during HIV infection. Curr. HIV/AIDS Rep..

[63] Kim P.S., Ahmed R. (2010). Features of responding T cells in cancer and chronic infection. Curr. Opin. Immunol..

[64] Day C.L., Kaufmann D.E., Kiepiela P., Brown J.A., Moodley E.S., Reddy S., Mackey E.W., Miller J.D., Leslie A.J., DePierres C. (2006). PD-1 expression on HIV-specific T cells is associated with T-cell exhaustion and disease progression. Nature.

[65] Barber D.L., Wherry E.J., Masopust D., Zhu B., Allison J.P., Sharpe A.H., Freeman G.J., Ahmed R. (2006). Restoring function in exhausted CD8 T cells during chronic viral infection. Nature.

[66] Ejrnaes M., Filippi C.M., Martinic M.M., Ling E.M., Togher L.M., Crotty S., von Herrath M.G. (2006). Resolution of a chronic viral infection after interleukin-10 receptor blockade. J. Exp. Med..

[67] Brooks D.G., Trifilo M.J., Edelmann K.H., Teyton L., McGavern D.B., Oldstone M.B. (2006). Interleukin-10 determines viral clearance or persistence *in vivo*. Nat. Med..

[68] Tinoco R., Alcalde V., Yang Y., Sauer K., Zuniga E.I. (2009). Cell-intrinsic transforming growth factor-beta signaling mediates virus-specific CD8+ T cell deletion and viral persistence *in vivo*. Immunity.

[69] Blackburn S.D., Shin H., Haining W.N., Zou T., Workman C.J., Polley A., Betts M.R., Freeman G.J., Vignali D.A., Wherry E.J. (2009). Coregulation of CD8+ T cell exhaustion by multiple inhibitory receptors during chronic viral infection. Nat. Immunol..

[70] Pellegrini M., Calzascia T., Toe J.G., Preston S.P., Lin A.E., Elford A.R., Shahinian A., Lang P.A., Lang K.S., Morre M. (2011). IL-7 engages multiple mechanisms to overcome chronic viral infection and limit organ pathology. Cell.

[71] Nanjappa S.G., Kim E.H., Suresh M. (2011). Immunotherapeutic effects of IL-7 during a chronic viral infection in mice. Blood.

[72] Cole G.A., Nathanson N., Prendergast R.A. (1972). Requirement for theta-bearing cells in lymphocytic choriomeningitis virus-induced central nervous system disease. Nature.

[73] Gilden D.H., Cole G.A., Nathanson N. (1972). Immunopathogenesis of acute central nervous system disease produced by lymphocytic choriomeningitis virus. II. Adoptive immunization of virus carriers. J. Exp. Med..

[74] Gilden D.H., Cole G.A., Monjan A.A., Nathanson N. (1972). Immunopathogenesis of acute central nervous system disease produced by lymphocytic choriomeningitis virus. I. Cyclophosphamide-mediated induction by the virus-carrier state in adult mice. J. Exp. Med..

[75] Kim J.V., Kang S.S., Dustin M.L., McGavern D.B. (2009). Myelomonocytic cell recruitment causes fatal CNS vascular injury during acute viral meningitis. Nature.

[76] Gresser I., Tovey M.G., Bandu M.E., Maury C., Brouty-Boye D. (1976). Role of interferon in the pathogenesis of virus diseases in mice as demonstrated by the use of anti-interferon serum. I. Rapid evolution of encephalomyocarditis virus infection. J. Exp. Med..

[77] Gresser I., Tovey M.G., Maury C., Bandu M.T. (1976). Role of interferon in the pathogenesis of virus diseases in mice as demonstrated by the use of anti-interferon serum. II. Studies with herpes simplex, Moloney sarcoma, vesicular stomatitis, Newcastle disease, and influenza viruses. J. Exp. Med..

[78] Gresser I., Maury C., Tovey M., Morel-Maroger L., Pontillon F. (1976). Progressive glomerulonephritis in mice treated with interferon preparations at birth. Nature.

[79] Gresser I., Tovey M.G., Maury C., Chouroulinkov I. (1975). Lethality of interferon preparations for newborn mice. Nature.

[80] Riviere Y., Gresser I., Guillon J.C., Tovey M.G. (1977). Inhibition by anti-interferon serum of lymphocytic choriomeningitis virus disease in suckling mice. Proc. Natl. Acad. Sci. U. S. A..

[81] Pfizenmaier K., Trostmann H., Rollinghoff M., Wagner H. (1975). Temporary presence of self-reactive cytotoxic T lymphocytes during murine lymphocytic choriomeningitis. Nature.

[82] Pfizenmaier K., Trostmann H., Rollinghoff M., Wagner H. (1976). Cell-mediated immunity in lumphocytic choriomeningitis. I. The specificity of the cytotoxic T lymphocytes. Z. Immunitatsforsch. Exp. Klin. Immunol..

[83] Ohashi P.S., Oehen S., Buerki K., Pircher H., Ohashi C.T., Odermatt B., Malissen B., Zinkernagel R.M., Hengartner H. (1991). Ablation of "tolerance" and induction of diabetes by virus infection in viral antigen transgenic mice. Cell.

[84] Oldstone M.B., Nerenberg M., Southern P., Price J., Lewicki H. (1991). Virus infection triggers insulin-dependent diabetes mellitus in a transgenic model: role of anti-self (virus) immune response. Cell.

[85] Evans C.F., Horwitz M.S., Hobbs M.V., Oldstone M.B. (1996). Viral infection of transgenic mice expressing a viral protein in oligodendrocytes leads to chronic central nervous system autoimmune disease. J. Exp. Med..

[86] Cornberg M., Sheridan B.S., Saccoccio F.M., Brehm M.A., Selin L.K. (2007). Protection against vaccinia virus challenge by CD8 memory T cells resolved by molecular mimicry. J. Virol..

[87] Chen H.D., Fraire A.E., Joris I., Brehm M.A., Welsh R.M., Selin L.K. (2001). Memory CD8+ T cells in heterologous antiviral immunity and immunopathology in the lung. Nat. Immunol..

[88] Welsh R.M., Che J.W., Brehm M.A., Selin L.K. (2010). Heterologous immunity between viruses. Immunol. Rev..

[89] Selin L.K., Nahill S.R., Welsh R.M. (1994). Cross-reactivities in memory cytotoxic T lymphocyte recognition of heterologous viruses. J. Exp. Med..

[90] Bergthaler A., Flatz L., Hegazy A.N., Johnson S., Horvath E., Lohning M., Pinschewer D.D. (2010). Viral replicative capacity is the primary determinant of lymphocytic choriomeningitis virus persistence and immunosuppression. Proc. Natl. Acad. Sci. U. S. A..

[91] Sullivan B.M., Emonet S.F., Welch M.J., Lee A.M., Campbell K.P., de la Torre J.C., Oldstone M.B. (2011). Point mutation in the glycoprotein of lymphocytic choriomeningitis virus is necessary for receptor binding, dendritic cell infection, and long-term persistence. Proc. Natl. Acad. Sci. U. S. A..

[92] Topalian S.L., Hodi F.S., Brahmer J.R., Gettinger S.N., Smith D.C., McDermott D.F., Powderly J.D., Carvajal R.D., Sosman J.A., Atkins M.B. (2012). Safety, activity, and immune correlates of anti-PD-1 antibody in cancer. N. Engl. J. Med..

[93] Brahmer J.R., Tykodi S.S., Chow L.Q., Hwu W.J., Topalian S.L., Hwu P., Drake C.G., Camacho L.H., Kauh J., Odunsi K. (2012). Safety and activity of anti-PD-L1 antibody in patients with advanced cancer. N. Engl. J. Med..

[94] Velu V., Titanji K., Zhu B., Husain S., Pladevega A., Lai L., Vanderford T.H., Chennareddi L., Silvestri G., Freeman G.J. (2009). Enhancing SIV-specific immunity *in vivo* by PD-1 blockade. Nature.

[95] Trautmann L., Janbazian L., Chomont N., Said E.A., Gimmig S., Bessette B., Boulassel M.-R., Delwart E., Sepulveda H., Balderas R.S. (2006). Upregulation of PD-1 expression on HIV-specific CD8+ T cells leads to reversible immune dysfunction. Nat. Med..

[96] Marshall H.D., Chandele A., Jung Y.W., Meng H., Poholek A.C., Parish I.A., Rutishauser R., Cui W., Kleinstein S.H., Craft J. (2011). Differential expression of Ly6C and T-bet distinguish effector and memory Th1 CD4(+) cell properties during viral infection. Immunity.

[97] Amanna I.J., Raue H.P., Slifka M.K. (2012). Development of a new hydrogen peroxide-based vaccine platform. Nat. Med..

[98] Flatz L., Cheng C., Wang L., Foulds K., Ko S.Y., Kong W.P., Roychoudhuri R., Shi W., Bao S., Todd J.P. (2012). Gene-based vaccination with a mis-matched envelope protects against simian immunodeficiency virus infection in non-human primates. J. Virol..

[99] Flatz L., Hegazy A.N., Bergthaler A., Verschoor A., Claus C., Fernandez M., Gattinoni L., Johnson S., Kreppel F., Kochanek S. (2010). Development of replication-defective lymphocytic choriomeningitis virus vectors for the induction of potent CD8+ T cell immunity. Nat. Med..

[100] Folk S., Steinbecker S., Windmeyer J., Macneil A., Campbell S., Rollin P.E. (2011). Lymphocytic choriomeningitis with severe manifestations, Missouri, USA. Emerg. Infect. Dis..

[101] Razonable R.R. (2011). Rare, unusual, and less common virus infections after organ transplantation. Curr. Opin. Organ. Transplant..

[102] Stahl J.P., Mailles A., Dacheux L., Morand P. (2011). Epidemiology of viral encephalitis in 2011. Med. Mal. Infect..

[103] Milazzo M.L., Campbell G.L., Fulhorst C.F. (2011). Novel arenavirus infection in humans, United States. Emerg. Infect. Dis..

[104] Grant-Klein R.J., Altamura L.A., Schmaljohn C.S. (2011). Progress in recombinant DNA-derived vaccines for Lassa virus and filoviruses. Virus Res..

[105] Lan S., McLay Schelde L., Wang J., Kumar N., Ly H., Liang Y. (2009). Development of infectious clones for virulent and avirulent pichinde viruses: A model virus to study arenavirus-induced hemorrhagic fevers. J. Virol..

[106] Albarino C.G., Bergeron E., Erickson B.R., Khristova M.L., Rollin P.E., Nichol S.T. (2009). Efficient reverse genetics generation of infectious junin viruses differing in glycoprotein processing. J. Virol..

[107] Flatz L., Bergthaler A., de la Torre J.C., Pinschewer D.D. (2006). Recovery of an arenavirus entirely from RNA polymerase I/II-driven cDNA. Proc. Natl. Acad. Sci. U. S. A..

[108] Popkin D.L., Teijaro J.R., Sullivan B.M., Urata S., Rutschmann S., de la Torre J.C., Kunz S., Beutler B., Oldstone M. (2011). Hypomorphic mutation in the site-1 protease Mbtps1 endows resistance to persistent viral infection in a cell-specific manner. Cell Host Microbe.

[109] Sanchez A.B., de la Torre J.C. (2006). Rescue of the prototypic Arenavirus LCMV entirely from plasmid. Virology.

[110] Popkin D.L., Teijaro J.R., Lee A.M., Lewicki H., Emonet S., de la Torre J.C., Oldstone M. (2011). Expanded potential for recombinant trisegmented lymphocytic choriomeningitis viruses: Protein production, antibody production, and *in vivo* assessment of biological function of genes of interest. J. Virol..

